# Cerebellar imaging for neuroscience at 9.4 T

**DOI:** 10.1002/mrm.30596

**Published:** 2025-06-28

**Authors:** W. van der Zwaag, D. H. Y. Tse, B. A. Poser, N. Priovoulos

**Affiliations:** ^1^ Spinoza Centre for Neuroimaging Royal Netherlands Academy for Arts and Sciences Amsterdam The Netherlands; ^2^ Computational and Cognitive Neuroscience and Neuroimaging, Netherlands Institute for Neuroscience Royal Netherlands Academy for Arts and Sciences Amsterdam The Netherlands; ^3^ Scannexus BV Maastricht The Netherlands; ^4^ Faculty of Psychology and Neuroscience Maastricht University Maastricht The Netherlands; ^5^ Department of Biomedical Engineering and Physics, Amsterdam University Medical Centers University of Amsterdam Amsterdam The Netherlands; ^6^ Oxford Centre for Integrative Neuroimaging, FMRIB, Nuffield Department of Clinical Neurosciences University of Oxford Oxford UK

**Keywords:** 3D EPI, cerebellum, MP2RAGE, MRI, ultra‐high field

## Abstract

**Purpose:**

This study explored the possibility of visualizing cerebellar structure and function in vivo with a 9.4 T protocol suitable for neuroscientific experiments.

**Methods:**

Six healthy individuals were scanned with a 9.4 T acquisition protocol including functional runs using BOLD‐weighted 3D EPI at 0.8 and 1.0 mm isotropic resolution, and a 0.4 mm MP2RAGE covering the entire cerebellum to derive cerebellar cortical surfaces.

**Results:**

Scan sessions took approximately 1 h, a duration generally well tolerated in (cognitive) neuroscience experiments. A generalized B_1_ shim over the cerebellum provided sufficient contrast for gray–white matter segmentation and surface generation in all participants. A motor‐task paradigm yielded consistent responses in both hemispheres in the posterior and anterior cerebellar lobes.

**Conclusion:**

These experiments show that it is feasible to undertake neuroscientific experiments in the human cerebellum using 9.4 T MRI. The increased SNR and BOLD sensitivity benefit both structural and functional acquisitions and derivatives such as cortical surfaces generated from these data.

## 1 INTRODUCTION

The cerebellum is a small and intricately shaped brain region located at the base of the skull. It is involved in brain functions ranging from motor control to language and cognition.^1^ Damage to the cerebellum can result in a correspondingly wide range of symptoms and is observed in different neurodevelopmental and neurological diseases.^2^ The cerebellar cortex is much thinner than the cerebral cortex (˜1 mm).^3^ To adequately visualize the intricate structure and function of the cerebellar cortical sheet requires spatial resolutions typically beyond the reach of in vivo MRI acquisitions^4^ as well as specific processing tools. Although targeted acquisitions at 7 T, for example, with anisotropic voxels have been used to visualize the cerebellar cortex in detail,^5^, ^6^ insufficient SNR remains a limiting factor for both structural and functional cerebellar imaging.

The term ultra‐high field (UHF) is typically used to describe all magnetic field strengths for human imaging at and above 7 T. UHF‐MRI offers both higher SNR^7^ and increased contrast sensitivity to BOLD for fMRI experiments.^8^, ^9^ Hence, UHF‐MRI is particularly sought‐after to investigate brain function at the mesoscopic scale.^10^ Although 7 T offers good BOLD sensitivity at submillimeter voxels, the sensitivity and SNR are expected to increase further at 9.4 T.^7^, ^11^

Although SNR increases and higher BOLD sensitivity both facilitate fMRI experiments at UHF,^12^ other aspects of increased field strength are less beneficial: as the wavelength of the RF pulses becomes shorter, the B_1_ transmit field inhomogeneity increases, leading to areas of very low signal in the resulting images. For example, the default CP mode of a 9.4 T whole brain transmit coil has a significant B_1_ drop out in the temporal lobes and the cerebellum,^13^ which hinders imaging.^6^ Parallel transmit approaches can be used to address or steer these inhomogeneities away from the area of interest.^14^, ^15^, ^16^ However, the acquisition of participant‐specific B_1_ information to calculate the B_1_ shim settings makes this a time‐consuming approach that is often incompatible with neuroscientific experiments. Recently, universal parallel transmit methods, in that calibration‐free RF settings optimized for a general population group are used, have been successfully demonstrated in several applications.^17^, ^18^, ^19^ A universal B_1_ shim would provide a simple and time‐saving approach to achieving cerebellum imaging without B_1_ dropouts at 9.4 T.

Typical neuroscientific acquisition sessions consist of one or more anatomical acquisitions with good gray–white matter contrast to delineate the cortex and other regions of interest, as well as time series data sensitive to brain function. At UHF, the MP2RAGE sequence is often used to acquire T_1_‐weighted scans with reduced sensitivity to B_1_ inhomogeneity.^20^ For functional acquisitions, BOLD contrast remains widely used, although several more spatially specific contrasts have been proposed.^21^, ^22^, ^23^ The 3D EPI is particularly attractive for submillimetre BOLD acquisitions at UHF. First, the intrinsic SNR advantage of 3D imaging at UHF outweighs its greater vulnerability to physiological noise compared to 2D EPI, making 3D EPI overall more sensitive for BOLD fMRI in these small voxels.^24^, ^25^ Second, the short excitation TR and hence use of small flip angles results in a drastically lower specific absorption rate (SAR) deposition, which can be a limiting factor for 2D EPI.

Here, we aimed to acquire high‐resolution cerebellar fMRI data with an acquisition protocol, comprising of a high‐resolution T_1_‐weighted MP2RAGE sequence to derive cerebellar cortical surfaces and multiple functional runs using BOLD‐weighted 3D EPI in scan sessions compatible with a neuroscientific study.

## 2 METHODS

All data were acquired on a 9.4 T human MR scanner (Siemens Healthineers) equipped with a head gradient set (AC84‐mk2, maximum amplitude 80 mT/m, maximum slew rate 333 T/m/s), a 16‐channel RF parallel transmission system (1 kW per channel) and a whole‐brain dual‐row 16‐channel transmit/31‐channel receive array coil.^13^ Six healthy individuals (two females, age range: 23–51 years) participated after providing informed consent, following approval of the research protocol from the local ethical committee at Maastricht University.

### 2.1 B_0_ and B_1_ optimization

The B_0_ field homogeneity at the cerebellum in each of the participants was optimized with a custom shimming routine. B_0_ field maps were obtained from a dual‐echo 3D gradient‐recalled echo sequence (TR = 30 ms, TE_1_ = 1.00 ms, TE_2_ = 3.21 ms, nominal flip angle = 11°, nominal voxel size = 4 mm isotropic, matrix‐size = 50 × 50 × 44, bandwidth = 1560 Hz/pixel, and T_acq_ = 1 min 49 s). B_0_ shimming, including all first and second order spherical harmonics as well as five higher order terms (Z^3^, Z^2^X, Z^2^Y, ZX^2^Y^2^, and Z^4^), was accomplished with an in‐house built software package.^26^

To reduce the overall scan time, increase participants' comfort and generate a protocol suitable for neuroscientific experimentation, a universal B_1_ shim approach was used in this study.^17^, ^27^ Previously acquired channel‐by‐channel complex B_1_ maps were used for the universal cerebellum B_1_ shim. These maps were measured in a separate cohort of five volunteers (two females, age range: 26–30 years) who did not participate in the cerebellar imaging sessions. The maps were obtained using a transmit phase‐encoded^28^ T_2_ and T_2_* compensated version of DREAM^29^ (TR_imaging train_ = 6.8 ms, TR = 7.5 s, TE_1_ = 2.22 ms, TE_2_ = 4.44 ms, nominal flip angle = 7°, nominal preparation pulse flip angle = 55.5°, imaging slice thickness = 4 mm, slice separation = 10 mm, preparation pulse slice thickness = 8 mm, voxel‐size = 4 mm isotropic, matrix‐size = 64 × 56 × 15, bandwidth = 690 Hz/pixel, 32 transmit phase encode steps, and T_acq_ = 4 min). In the same cohort, B_0_ maps were measured, same as described above.

To calculate the universal B_1_ shim, cerebellar masks were first generated by projecting the cerebellar region of interest (ROI) of the Montreal Neurological Institute (MNI) atlas to individual‐level through registering the echo‐combined magnitude images from the B_0_ maps to the MNI152 template using FSL's FLIRT.^30^ The cerebellum‐masked channel‐by‐channel complex B_1_ maps from the five individuals were concatenated to form the system matrix of the optimization. The universal B_1_ shim for the cerebellum was calculated using a magnitude‐least‐square method^31^ optimized with a parallel conjugate–gradients‐based algorithm.^32^ A phase‐only universal B_1_ shim was used. The universal B_1_ shim was applied to the five individual channel‐by‐channel complex B_1_ maps to simulate the shim results and to obtain an averaged scaling factor for the scanner's reference transmit voltage. The flip angle achieved by the CP mode and the universal B_1_ shim were mapped in one extra participant using a pre‐saturation turbo‐flash sequence^33^ with parameters: TR_imaging train_ = 5.9 ms, TR = 10s, TE = 2.24 ms, nominal flip angle = 8°, nominal preparation pulse flip angle = 90°, imaging slice thickness = 4 mm, voxel‐size = 4 mm isotropic, matrix‐size = 64 × 64 × 15, bandwidth = 690 Hz/pixel, and T_acq_ = 20 s.

### 2.2 Neuroscientific acquisitions

For the neuroscientific experiments, a 0.4 mm isotropic resolution MP2RAGE slab was first acquired covering the entire cerebellum similar to 7 T acquisitions used previously^6^ (TR_MP2RAGE_ = 5500 ms, TR_FLASH_ = 7.5 ms, TE = 3.66 ms, flip angles = 9°/6°, TI_1_/TI_2_ = 1000/2900 ms, matrix‐size = 520 × 310 × 160, FOV = 210 × 124 × 64 mm^3^, bandwidth = 250 Hz/Px, and T_acq_ = 16 min 10 s).

A 1 mm isotropic resolution 3D EPI was acquired during a motor task with an axial‐oblique FOV = 186 × 186 × 60 mm^3^ covering the cerebellum (TR = 42 ms, TE = 19 ms, flip angle = 10°, TR_volume_ = 2520 ms, EPI bandwidth = 1852 Hz/Px, and matrix‐size = 180 × 180 × 60). GRAPPA = 3 and phase partial‐Fourier = 7/8 were used to limit TE to ˜T_2_* of gray matter in the cerebellum. A total of 120 timepoints were collected in the T_acq_ (5 min 15 s). The motor task alternated between 11 s REST and ON blocks during which all fingers of the right hand were moved. Visual task instructions were provided on a screen positioned at the back of the RF‐coil.

Three‐dimensional EPI scans at 0.8 mm isotropic resolution were also acquired during a motor task with an axial‐oblique FOV = 186 × 186 × 48 mm^3^ covering most of the cerebellum (TR = 49 ms, TE = 22 ms, flip angle = 12°, TR_volume_ = 2940 ms, EPI bandwidth = 1540 Hz/Px and matrix‐size = 232 × 232 × 60). GRAPPA = 4 and phase partial‐Fourier = 7/8 were similarly used to limit TE to ˜T_2_* of gray matter. A total of 164 timepoints were collected in the T_acq_ (8 min 18 s). The same motor task was used as for the 1 mm runs.

### 2.3 Analysis

An in‐house developed Nighres algorithm was used for the automated segmentation of the cerebellar cortex.^34^, ^35^ The segmentations were visually inspected and densely tessellated to a mesh. The 3D EPI time series were motion‐corrected, generalized linear model were fitted (stimulus > rest) using the FMRI Expert Analysis Tool (FEAT; FSL 6.0.3)^36^ and EPI‐to‐MP2RAGE linear transforms were calculated using the Advanced Normalization Tools (ANTs) 2.1.1.^37^

For one participant, an approximately matched 0.4 mm isotropic MP2RAGE and 3D‐EPI slab was available from a cerebellar imaging study performed at 7 T from the authors (7 T acquisition details^6^, ^38^). The derived T_1_ maps and segmentations were compared visually as an informal cross‐field comparison of data quality. From the segmentations, adjacent gray and white matter ROIs were defined. These ROIs were used to compare T_1_ values, SNR and contrast‐to‐noise (CNR). SNR was defined as ${\mathrm{SNR}}_{\mathrm{WM},\mathrm{GM}}=\frac{\mu_{\mathrm{WM},\mathrm{GM}}}{\sigma_{\mathrm{WM},\mathrm{GM}}}$ with μ: mean T_1_ value within ROI and σ: SD within ROI and CNR was defined as $\mathrm{CNR}=\frac{\mathrm{abs}\left(\mu_{\mathrm{WM}}-\mu_{\mathrm{GM}}\right)}{\sqrt{\sigma_{\mathrm{WM}}^{2}+\sigma_{\mathrm{GM}}^{2}}}$. The cross‐field 3D‐EPIs were visually compared against the MP2RAGE for spatial distortions.

## 3 RESULTS

Scan sessions took 55 to 65 min and were well tolerated by all participants.

**FIGURE 1:**
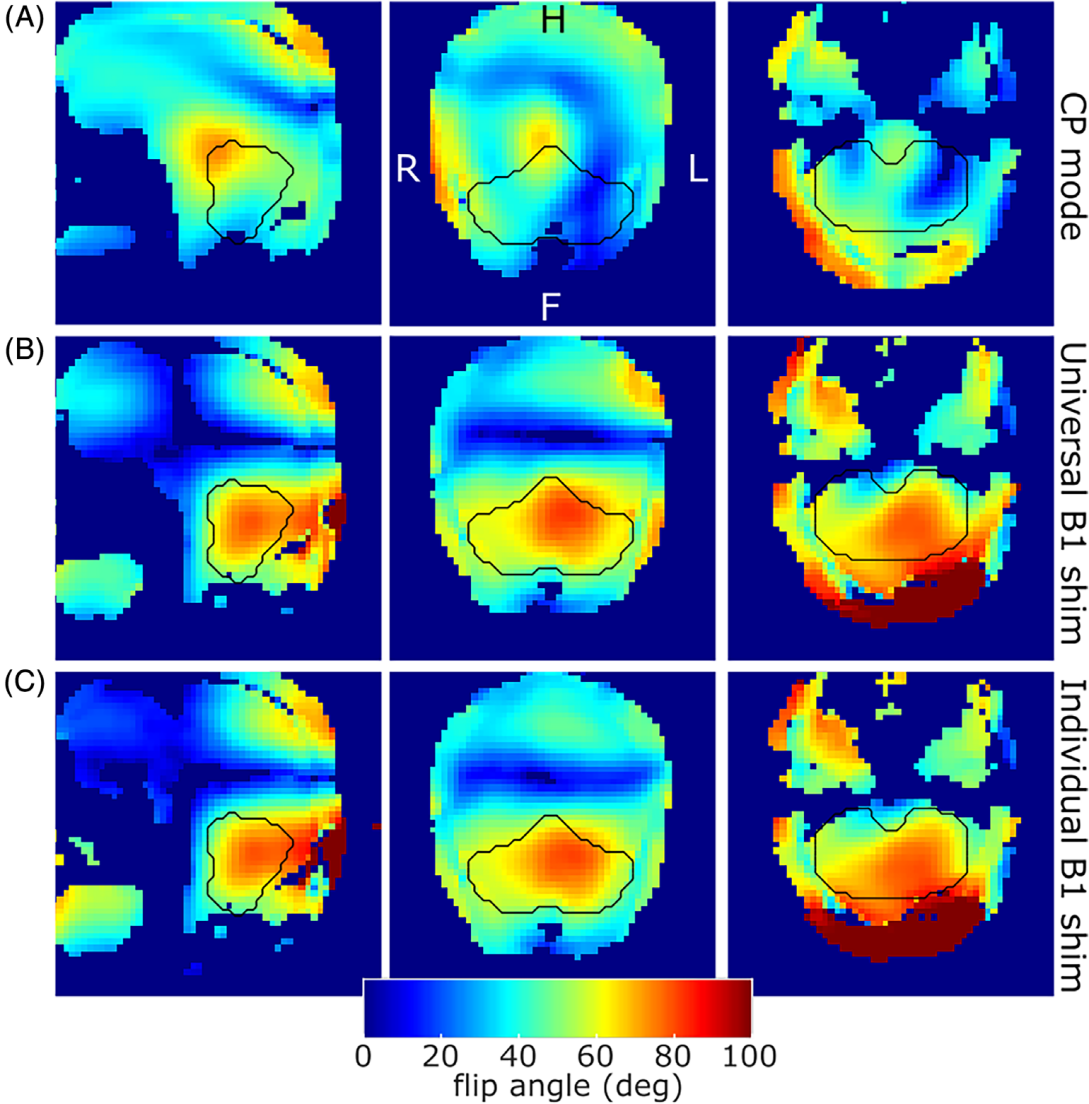
Pre‐saturation turbo‐flash (PreSat‐TFl) flip angle maps for CP mode, universal and individual B_1_ shims. The black lines outline the cerebellar mask used in the individual B_1_ shim. The target flip angle of the pre‐saturation pulse in the protocol was set to 90° and the mean flip angle achieved with the universal B_1_ shim in the cerebellum was approximately 60° (middle row), comparable to the individual shim (bottom row).

The higher‐order, cerebellum‐specific B_0_ shimming greatly reduced B_0_ variation over the cerebellum, resulting in predicted ΔB_0_ distributions similar to those achieved at lower field strengths (Figure S1, 9.4 T group range of ΔB_0_ SD = [30.47–56.86] Hz vs. reported 7 T values ˜50 Hz^39^). The universal B_1_ shims provided sufficient B_1_ efficiency and homogeneity in the cerebellum. In the single‐subject test of universal B_1_ shim versus personalized B_1_ shim, the universal B_1_ shim results were of a level similar to the individually optimized B_1_ shims, with comparable flip angles achieved throughout the cerebellar ROI for both B_1_ shim settings (Figure 1). For both universal and individual B_1_ shims, the achieved flip angle was approximately 60° for a 90° target. Consequently, a scaling factor of 1.5 was used in cerebellum imaging protocols for all flip angles.

**FIGURE 2:**
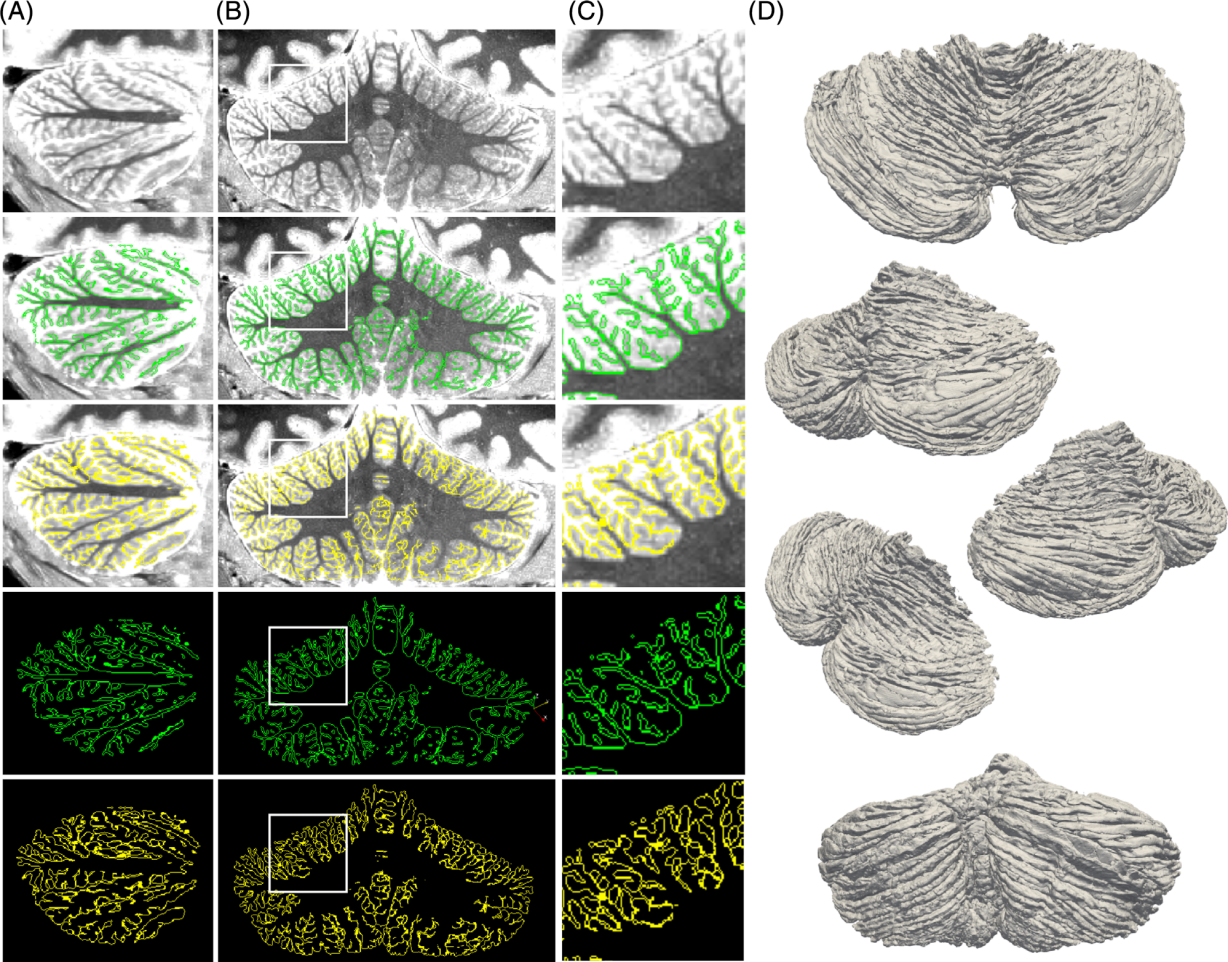
(A) Sagittal, (B) coronal, and (C) cropped views of the T_1_ map (top row) and the white and gray matter surfaces overlaid (top‐middle) and on their own. Individual folia were consistently segmented. (D) Various views of the cerebellar cortical surface reconstruction. Note the consistent ribbed‐like appearance, representing the cerebellar fissures.

On visual inspection, good contrast was achieved between cerebellar white and gray matter throughout the cerebellum in the MP2RAGE data of all individuals. Figure S2 highlights the consistency of image contrast achieved for heterogeneous cerebellar anatomies. None of the images presented with obvious B_1_ artifacts, which would appear in MP2RAGE data as areas with low contrast and high signal intensity.^40^ This allowed the successful segmentation of the cerebellar white and gray matter, up to the level of individual fissures (Figure 2). The morphological details in the 3D‐reconstructed cerebellar cortical surface, including the reconstruction of individual fissures giving the cerebellar surface a characteristic ribbed appearance, confirmed the anatomical validity of the segmentation (Figure 2D).

The median T_1_ value across participants in the cerebellar gray matter at 9.4 T was 2554 ms (interquartile range [IQR] = [2483–2624] ms), whereas for white matter the median T_1_ value across participants was 2124 ms (IQR = [1917–2331] ms).

**FIGURE 3:**
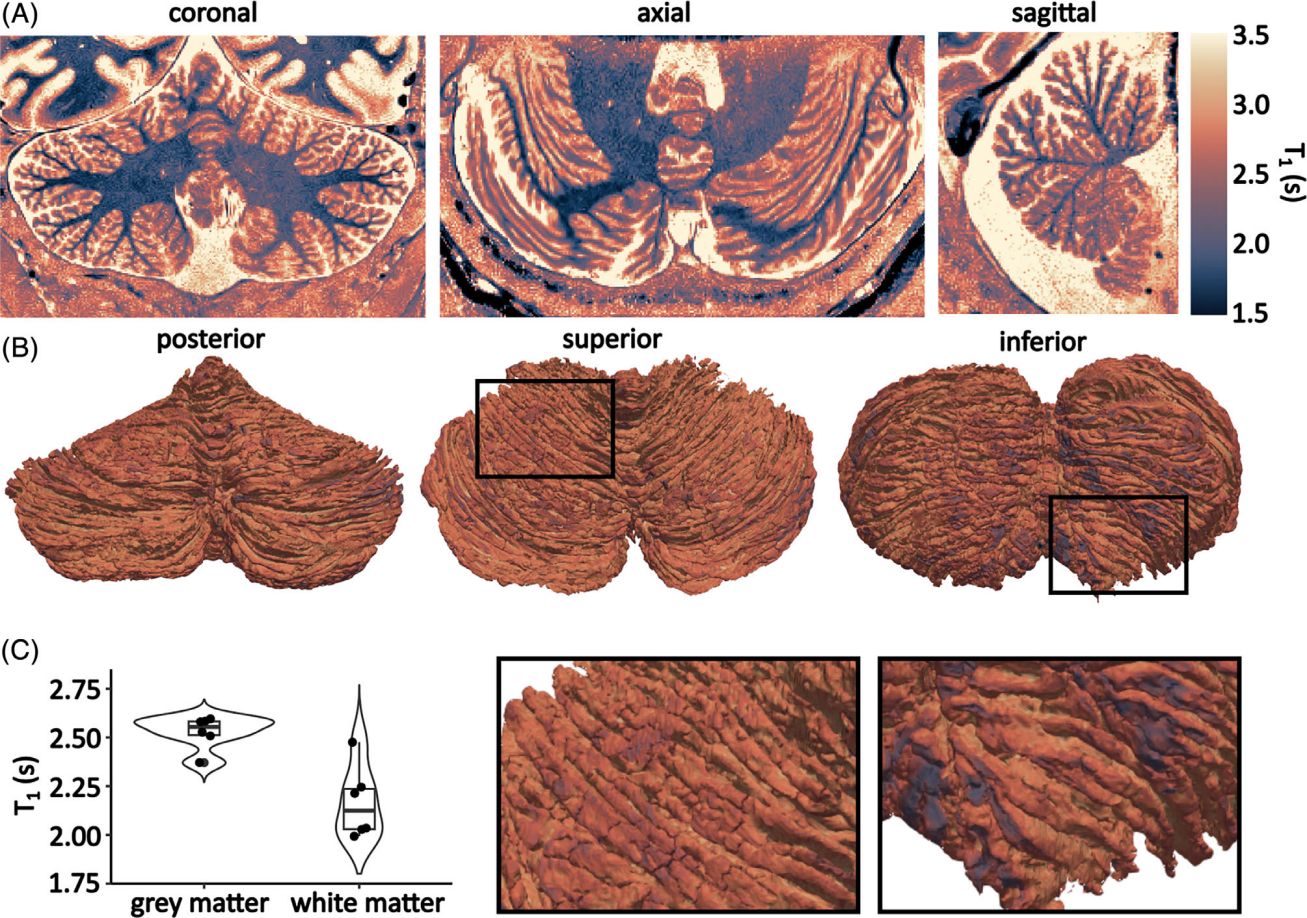
(A) T_1_ map derived from the MP2RAGE acquisition for one example participant. (B) T_1_ map projection on the cerebellar cortical surface and cropped views (black boxes, bottom). (C) Median gray matter and white matter cerebellar T_1_ estimates.

Projections of the T_1_ values onto the cerebellar surfaces of the individual participants, as shown in Figure 3B and Figure S3, show consistent T_1_ values throughout. This suggests partial volume effects in the segmentations to be limited, despite the highly foliated surface.

**FIGURE 4:**
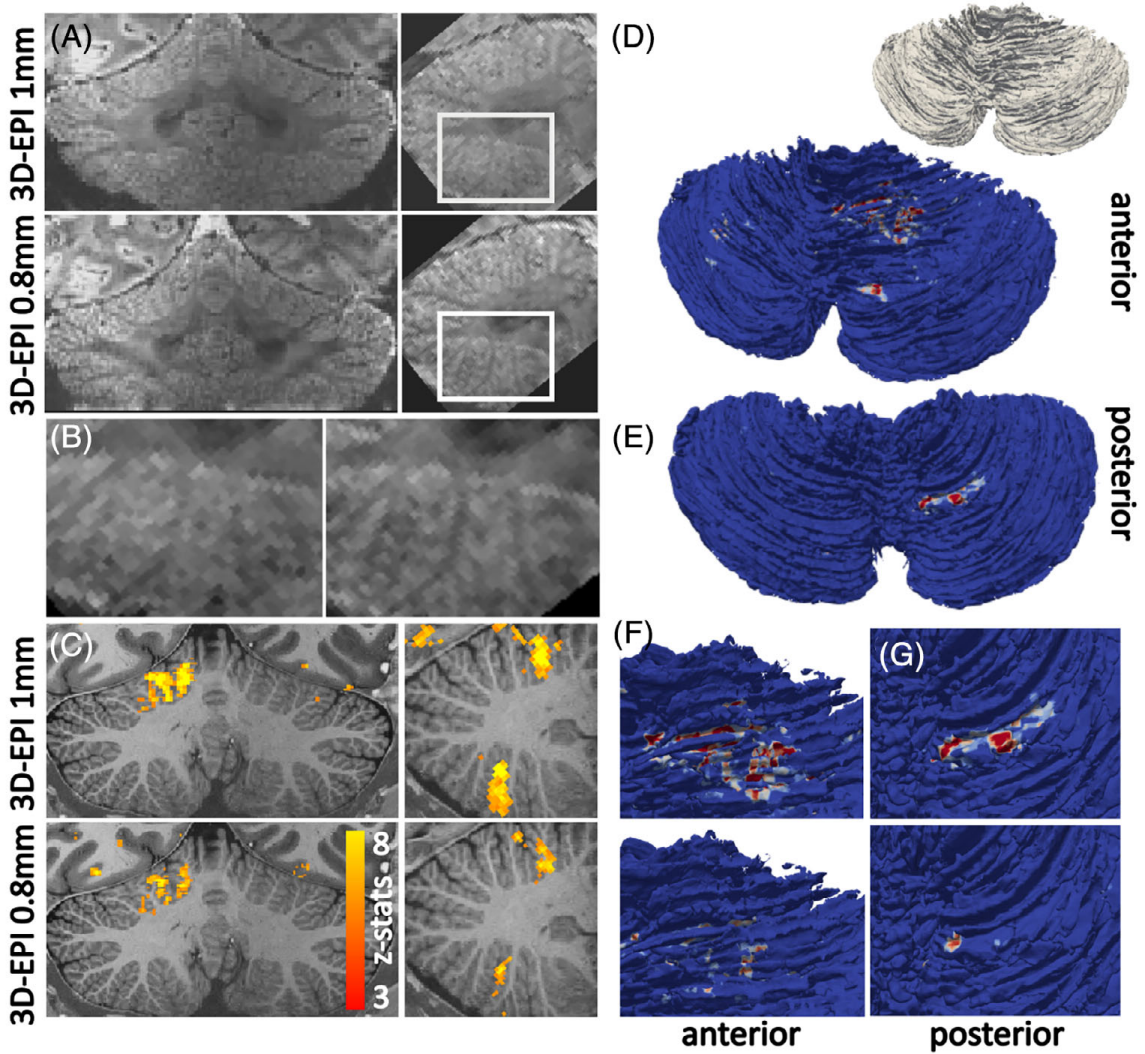
(A) Example coronal (left) and sagittal (right) mean 3D EPIs at the cerebellar level. Top, 1 mm; bottom, 0.8 mm nominal resolution. (B) Zoomed view of the cerebellum. Note that folia details can be teased apart in the 0.8 mm 3D EPI. (C) Activation maps (z‐stats) for the finger‐tapping task with the 1 mm 3D EPI (top) and the 0.8 mm 3D EPI (middle) and anatomical reference in the bottom. Note that clusters were observed both in the posterior and anterior lobes of the cerebellum, suggesting adequate B_1_ homogeneity. (D–G) Projection of fMRI z‐stats on cortical surface for the same participant. (D) z‐stat projection on individual cerebellar surface for 1 mm acquisition (anterior view) and posterior view (E). (F) Zoomed views of the anterior lobe motor cluster for 1 mm (top) and 0.8 mm (bottom). (G) Zoomed view of the posterior lobe motor cluster for 1 mm (top) and 0.8 mm (bottom).

Both the 1 mm and 0.8 mm 3D EPI protocols showed good image quality, with homogeneous image brightness throughout the cerebellum (Figure 4A) and visible cerebellar foliations in the mean EPI data (Figure 4B). The employed 3D‐EPI acquisition protocols and B_0_ shimming resulted in limited spatial distortions, as ascertained visually against the outline of the cerebellum from structural imaging. These distortions were similar to those encountered at 7 T in an informal comparison with data from the same participant and a similar acquisition slab and readout length/echo spacing (Figure S4). fMRI responses to the finger‐tapping task were detectable following a single‐acquisition run (Figure 4C). The consistency of the detection of these responses across participants is demonstrated in Figure S5. fMRI responses were detected in both the anterior and posterior lobe (Figure 4C–G), in accordance with the known neuronal responses in the human cerebellum.^41^ This balance between responses in the anterior and posterior lobe suggests that B_1_ inhomogeneity was appropriately tackled in the fMRI acquisition as well as in the MP2RAGE.

To facilitate visual inspection and surface‐based analysis, fMRI results are often projected onto a (cortical) surface. Here, we demonstrate the feasibility of this with cerebellar fMRI data. Projections of the fMRI z‐maps onto the cerebellar cortical surface are shown for a representative participant in Figure 4D–G. The spatial extent of the responses in the 1 mm and 0.8 mm data were comparable between the anterior and posterior lobe of the cerebellum.

**FIGURE 5:**
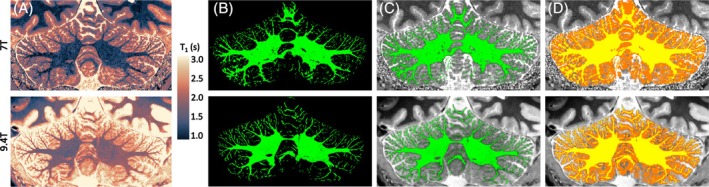
(A) Approximate comparison of 7 T versus 9.4 T T_1_ maps from an MP2RAGE for the same participant (top row: 7 T; bottom row: 9.4 T; coronal slice). Although accurate cross‐field comparisons are challenging, the 9.4 T SNR gain is visually obvious. (B) White matter segmentation, overlaying the anatomy (C). (D) White and gray matter segmentation overlaying the anatomy. The 9.4 T data produce segmentations with improved fidelity to the underlying anatomy.

Finally, we compared the T_1_ maps and segmentation results obtained in a single individual at 7 T and at 9.4 T, using approximately matched acquisitions and the same data‐processing approach (Figure 5). Compared to 7 T, the 9.4 T MP2RAGE T_1_ map shows higher SNR and CNR between white and gray matter (Table S1). T_1_ values were longer in both gray and white matter at the 9.4 T, as expected.^42^

At this resolution, the 7 T segmentations show visible noise, for example, holes within the main mass of WM and biologically implausible thickening in the right hemisphere gray matter. The 7 T segmentations visibly track less cortical detail, especially the small cortical branches in the right cerebellar hemisphere, which indicates a B_1_‐inhomogeneity driven effect. The 9.4 T segmentations instead show continuous delineations and create cortical surfaces that show the expected cerebellar anatomical characteristics, such as a coherent pattern of fissures in the mediolateral direction and an overall symmetric gray matter volume distribution (Figure 2; also compare with Figure 4 in Priovoulos et al.^6^). This is in line with the measured higher SNR at 9.4 T compared to 7 T.

Note that, although the acquisitions for these scans were matched in terms of resolution and field of view, the hardware including the field strength and RF‐coil design were different, leading to different B_1_‐performance, although a similar B_1_ shimming approach was used in both datasets. Interestingly, the 7 T dataset shows an area of low contrast and erroneously large white matter compartments in the right posterior cerebellar hemisphere, which is absent in the 9.4 T data. This is in line with the expected improvement in regional B_1_ shimming, because of (1) the more localized transmit profile from each of the coil elements, due to the increased field strength and smaller individual coils; and (2) because of the high number of transmit elements (16 channels), which provides additional degrees of freedom.

## 4 DISCUSSION

Advancing functional and anatomical MRI to higher spatial resolutions is crucial for understanding the mesoscale structure and function of the human brain. This goal can, in theory, be achieved by increasing the B_0_ field and leveraging the resulting SNR gain. In practice, however, few neuroscientific studies have used the sensitivity available at B_0_ >7 T, partly because of the inherent acquisition complexities.

In this study, we aimed to demonstrate that efficient imaging sessions, compatible with neuroscientific experiments in terms of acquisition and preparation time, are feasible at 9.4 T. To this end, we acquired fMRI data and T_1_‐weighted anatomical images, specifically of the human cerebellum. The cerebellum can serve as an effective testbed for human UHF neuroimaging technology, as it benefits from the increased SNR, while also presenting UHF‐related challenges, like B_1_ and B_0_ inhomogeneities. Our experiments showed that SAR limitations, B_1_ and B_0_ inhomogeneity can be effectively managed in the context of a typical neuroscientific experiment.

Excellent T_1_‐contrast homogeneity was achieved through a combination of a bias‐field insensitive MP2RAGE^20^ sequence (widely used at 7 T) and B_1_ shimming using a universal shim. The MP2RAGE uses a single inversion pulse with two readout‐trains and is by nature not SAR‐intensive. For the functional MRI acquisitions we used a 3D EPI sequence that, because of the short TR, yields highest SNR at modest flip angles, while enabling submillimetre resolutions.^24^, ^25^

At UHF, the CP‐mode of a typical whole‐head coil provides only limited B_1_ coverage in the cerebellum. At 9.4 T, a B_1_ void can be seen in the CP‐mode flip angle map in the cerebellum (Figure 1), making it impossible to image the whole cerebellum. We were able to shift the B_1_ “hot spot” by a phase‐only B_1_ shim to cover the whole cerebellum relatively uniformly. This allowed imaging of the entire cerebellum. We also found that it was sufficient to use a previously calculated universal shim, hence saving approximately 10 min per scan session. However, if imaging of other brain regions or the whole brain is required simultaneously, then full parallel transmit RF pulse optimization, such as kt‐points, would be necessary.

The main motivation behind using higher field strength for fMRI is the increased sensitivity to within‐voxel field inhomogeneities that create the deoxyhemoglobin‐dependent BOLD contrast. This sensitivity to field inhomogeneities, however, also leads to both increased within‐voxel signal dephasing and increased spatial distortions. Such artifacts are particularly pronounced in the lower brain, including the cerebellum, because of the proximity of the imaging slab to brain tissue/air interfaces. In practice, we found that a previously established static shim approach^27^ adequately mitigated such artifacts, resulting in robust fMRI responses in large slabs at submillimetre resolution.

Note that at the highest spatial resolution of 0.8 mm isotropic acquisition, ripple‐like artifacts were evident (Figure 4A,B) that are likely to decrease fMRI sensitivity. There are several potential reasons for these artifacts: the necessarily slightly increased TE compared to the 1 mm isotropic slab also increased the sensitivity to remaining static macroscale field inhomogeneities. More likely, it reflects phase inhomogeneities between acquisition shots: the combination of the necessarily increased segmentation factor and higher field strength results in an increased sensitivity to respiration‐related dynamic ΔB_0_.^25^ Furthermore, in practice the EPI k‐space trajectories are imperfect. At 7 T and 11.7 T field strengths, these imperfections were recently linked to eddy currents because of the coupling of third order B_0_ shims and readout gradients from the same vendor as used here.^43^ Such imperfections would create accumulative effects with longer k‐space trajectories and hence higher spatial resolutions. In practice, these artifacts were not prohibitive for successful fMRI acquisition at the challenging region of the human cerebellum. Results can likely be improved by tackling the eddy currents, adjusting the acquisition strategy (e.g., dual‐polarity EPI) and shot‐to‐shot B_0_ measurements and subsequent correction during the reconstruction.^44^, ^45^, ^46^ Addressing such issues is likely necessary to also unlock more complex cognitive tasks at UHF, such as those requiring natural motion.

One final concern with B_0_ >7 T is the participant reaction to the magnetic environment itself. In line with previous studies,^47^ we can report that none of the participants in this cohort (*n* = 6, experienced MRI participants albeit at lower field strengths) reported any long‐lasting effects.

Functional MRI stands to benefit significantly from ultra‐high field, especially for the study of more subtle cognitive responses in small size cohorts, where individual‐based analyses are preferential.^9^, ^48^ The finger‐tapping task used here is known to lead to reproducible results even at lower fields.^38^ Nevertheless, the short acquisition times used for the functional runs (˜5 min) and the overall limited session duration leave ample time for inclusion of additional or longer runs in a single session, which would still be acceptable to most participants in cognitive neuroscience experiments.

Animal literature indicates that the cerebellar cortex has a fine pattern of neuronal clustering and coordinated involvement, ranging from mediolateral zones and anterior–posterior stripes^49^, ^50^ up to patch‐like responses along the parallel fibers.^51^ This functional organization is underexplored in humans, because of resolution limitations in the current in vivo acquisition and analysis methodology.^4^, ^35^ However, human multi‐domain task and resting state mapping indicate a finer organization beyond the gross lobular level^1^, ^52^, ^53^, ^54^, ^55^, ^56^ with high spatial‐resolution, high‐quality data in individual participants better predicting individual boundaries.^52^ Recent progress in UHF structural and fMRI acquisition methods at 7 T^57^, ^58^ has helped push the spatial resolution, allowing the exploration of the cerebellar laminar organization^5^, ^6^, ^59^ or finely structured digit responses^38^, ^60^, ^61^ or others^62^, ^63^ in vivo. Nevertheless, it is estimated that fully resolving the human cerebellar cortex requires further pushing the spatial resolution, possibly up to the level of 0.2 mm isotropic,^4^ necessitating a matching increase in SNR. The methodology we describe here, combining high functional and anatomical spatial resolution at B_0_ = 9.4 T with appropriate processing, can help resolve the cerebellar functional response closer to the level of individual folia. Further pushing the UHF envelope^43^, ^64^, ^65^ has the potential to greatly increase our understanding of the functional organization of the human cerebellum.

## 5 CONCLUSION

In this work, we showed that neuroscientific MRI applications in the human cerebellum, using routine procedures, are feasible at 9.4 T and promise more detailed insights into its function and structure than what has been seen at 7 T. Despite ongoing challenges in hardware and B_1_ management, this presents an exciting prospect for what may be achieved at even higher field strengths, which are now becoming available for human imaging.

## CONFLICT OF INTEREST STATEMENT

Desmond Tse is an employee of Scannexus, a private company based in the Netherlands.

## Supporting information


**Figure S1.** Measured and predicted Δ*B*_0_ maps (left column) and histograms (right column) across individuals.
**Figure S2.** *T*_1_ maps for different views (columns) and participants (rows).
**Figure S3.** Projection of the *T*_1_ maps to the cerebellar cortical surface for different views (columns) and participants (rows).
**Figure S4.** Mean images of 3D‐EPI slabs at 9.4 T for one participant against *T*_1_ map (top) and approximately‐matching 3D‐EPI at 7 T (bottom). The anatomical delineation of the cerebellum is shown in orange. The arrows indicate the first phase‐encoding direction (PE_1_), that is the direction in which spatial distortions would be primarily expected.
**Figure S5.** fMRI responses for each participant (columns). (A) MP2RAGE slice. (B) mean 3D EPI 1 mm slice. (C) mean 3D EPI 0.8 mm slice. (D) fMRI response (z‐stats) for hand flexing using the 3D EPI 1 mm protocol. (E) zoomed fMRI response in posterior lobe. (F) fMRI response (*z*‐stats) for hand flexing using the 3D EPI 0.8 mm protocol. (G) zoomed fMRI response in posterior lobe.
**Table S1.** *T*_1_ values, signal‐to‐noise ratio and contrast‐to‐noise ratio between white (WM) and gray matter (GM) cerebellar ROIs.
