# Supplementary material for: Cerebellar imaging for neuroscience at 9.4 T
Source: Magn Reson Med. 2025 Jun 28;94(5):2129–39. doi: 10.1002/mrm.30596 (PMC12393186; doi:10.1002/mrm.30596)
Supplement: Supplementary file 1 — Figure S1. Measured and predicted ΔB 0 maps (left column) and histograms (right column) across individuals. Figure S2. T 1 maps for different views (columns) and participants (rows). Figure S3. Projection of the T 1 maps to the cerebellar cortical surface for different views (columns) and participants (rows). Figure S4. Mean images of 3D‐EPI slabs at 9.4 T for one participant against T 1 map (top) and approximately‐matching 3D‐EPI at 7 T (bottom). The anatomical delineation of the cerebellum is shown in orange. The arrows indicate the first phase‐encoding direction (PE1), that is the direction in which spatial distortions would be primarily expected. Figure S5. fMRI responses for each participant (columns). (A) MP2RAGE slice. (B) mean 3D EPI 1 mm slice. (C) mean 3D EPI 0.8 mm slice. (D) fMRI response (z‐stats) for hand flexing using the 3D EPI 1 mm protocol. (E) zoomed fMRI response in posterior lobe. (F) fMRI response (z‐stats) for hand flexing using the 3D EPI 0.8 mm protocol. (G) zoomed fMRI response in posterior lobe. Table S1. T 1 values, signal‐to‐noise ratio and contrast‐to‐noise ratio between white (WM) and gray matter (GM) cerebellar ROIs. [file MRM-94-2129-s001.docx]

**Supplementary material:**

Figure S1: Measured and predicted ΔB_0_ maps (left column) and histograms (right column) across individuals.


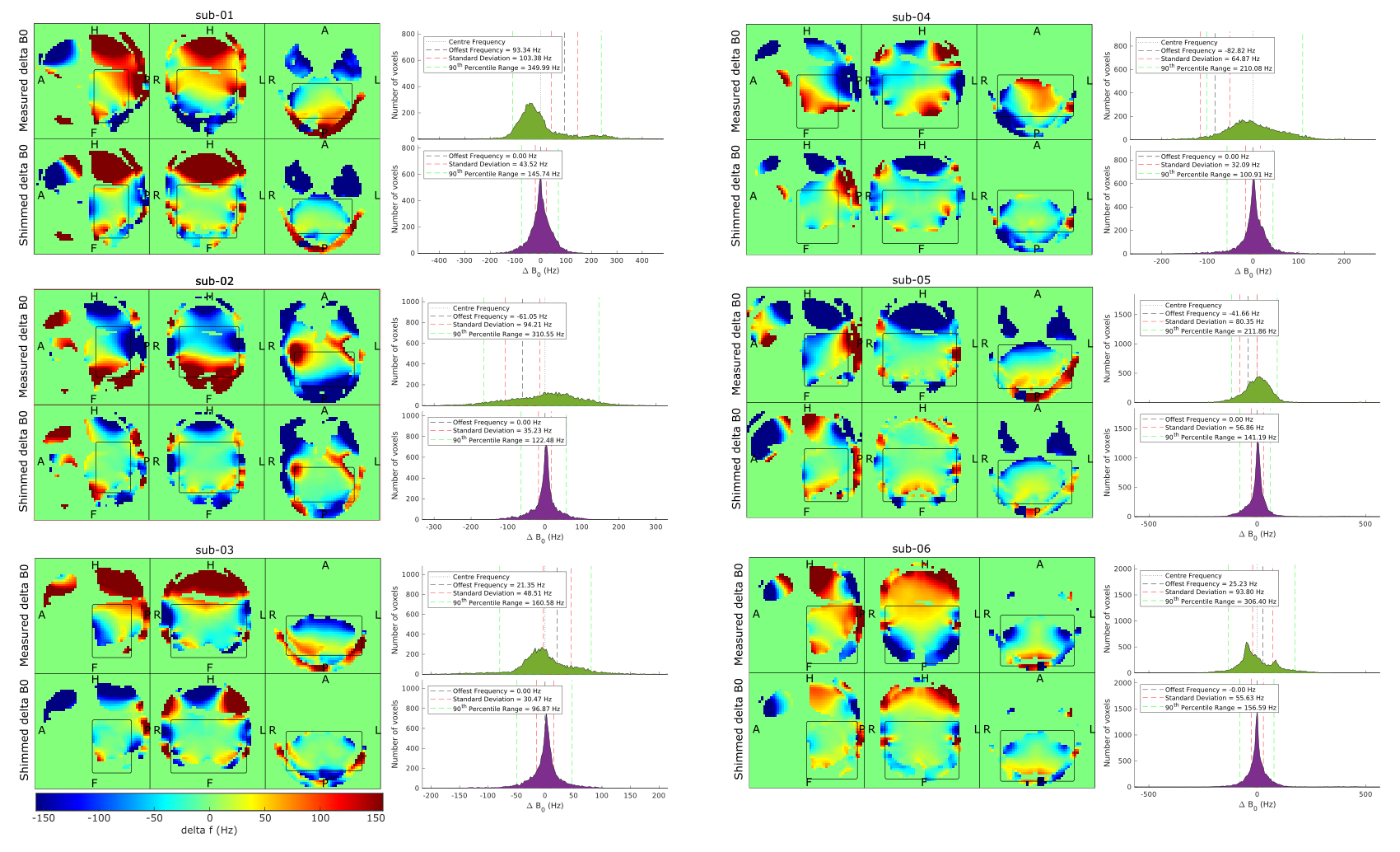


Figure S2: T_1_ maps for different views (columns) and participants (rows).
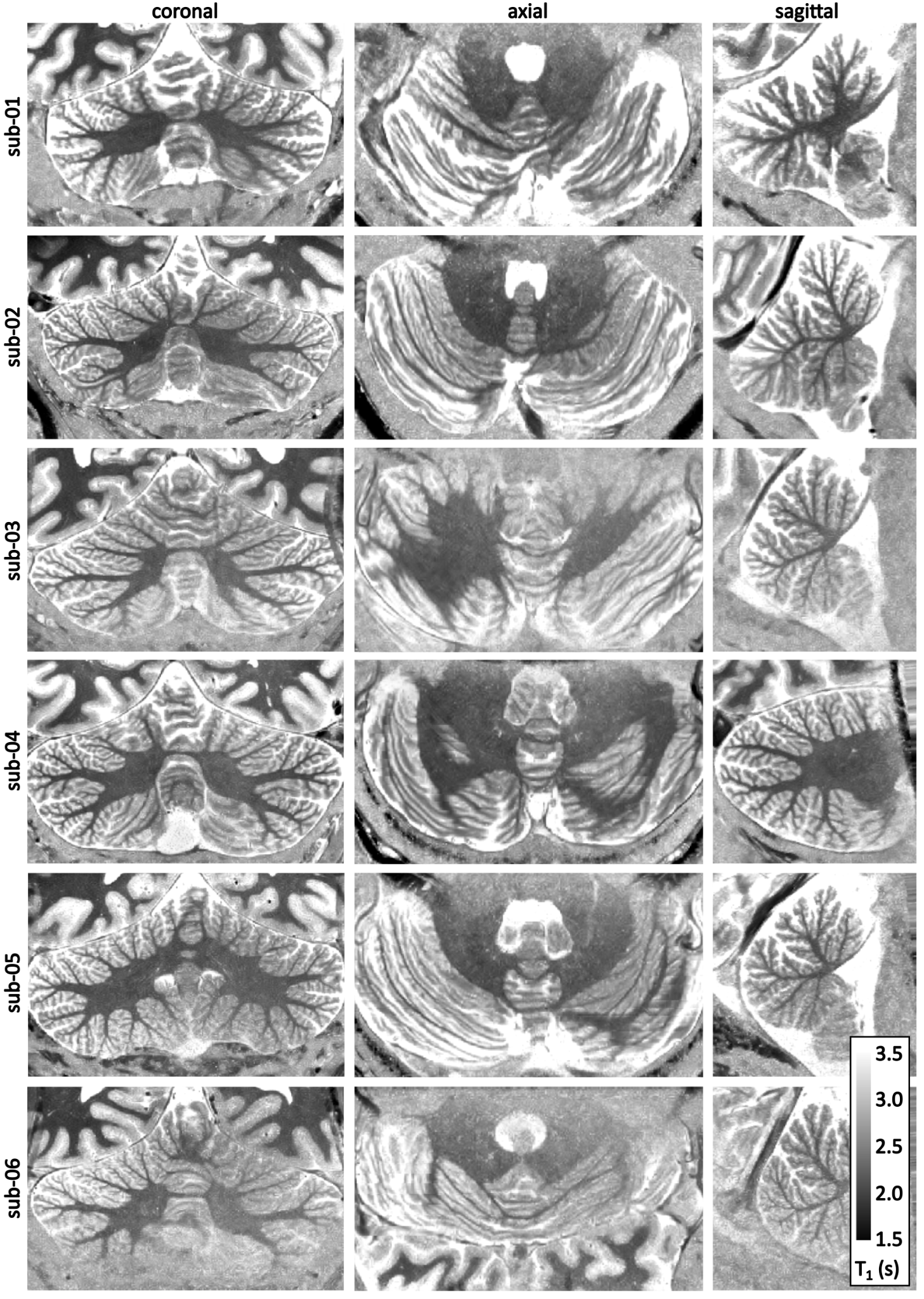


Figure S3: Projection of the T_1_ maps to the cerebellar cortical surface for different views (columns) and participants (rows).
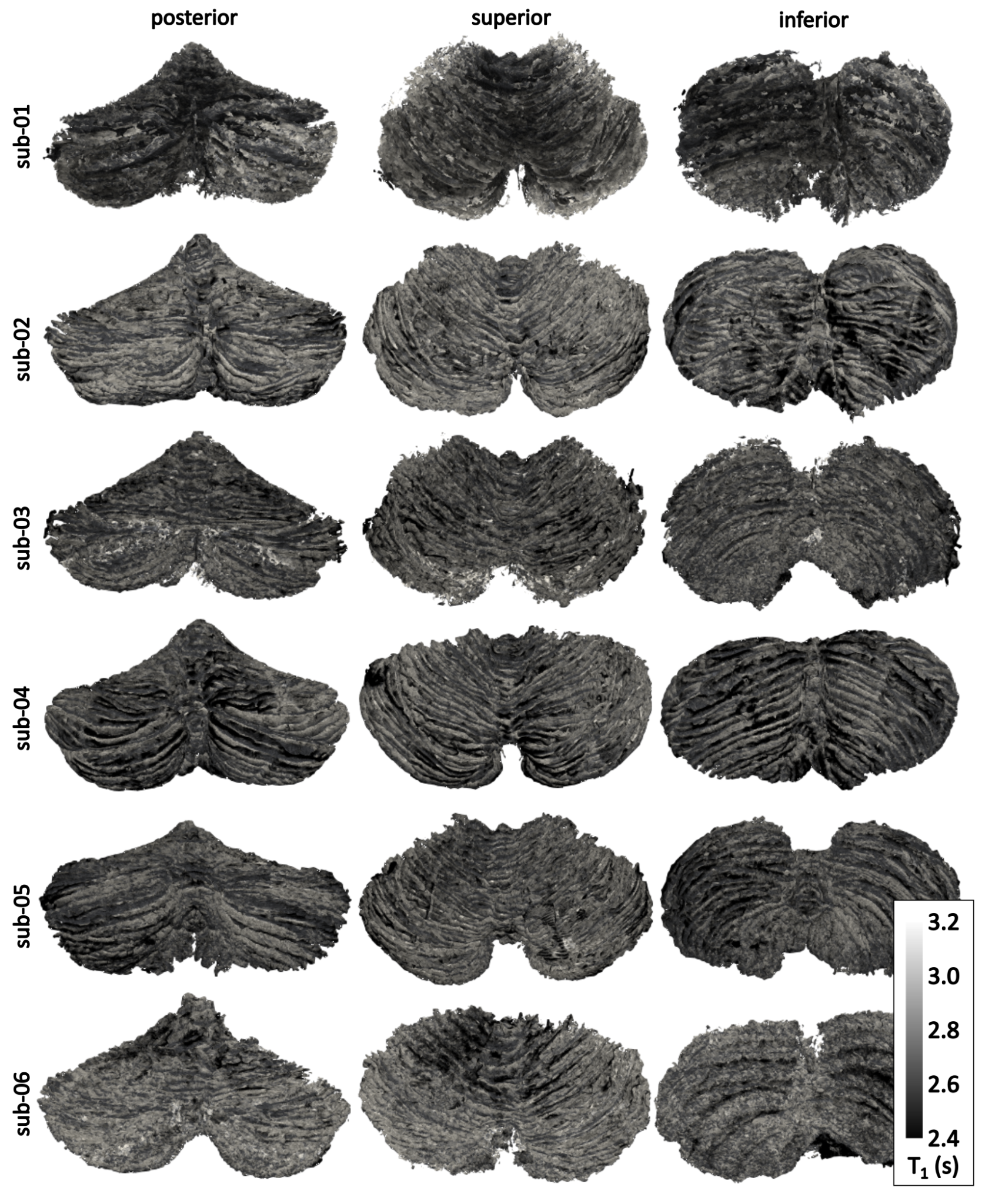


Figure S4: Mean images of 3D-EPI slabs at 9.4T for one participant against T_1_ map (top) and approximately-matching 3D-EPI at 7T (bottom). The anatomical delineation of the cerebellum is shown in orange. The arrows indicate the first phase-encoding direction (PE_1_), i.e. the direction in which spatial distortions would be primarily expected.


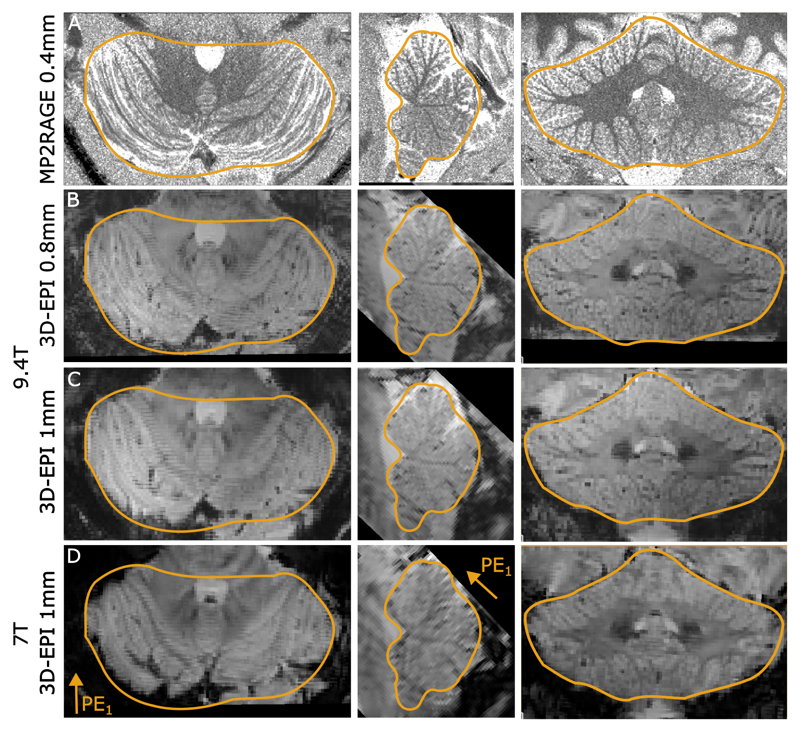


Figure S5: fMRI responses for each participant (columns). A, MP2RAGE slice. B, mean 3D EPI 1mm slice. C, mean 3D EPI 0.8mm slice. D, fMRI response (z-stats) for hand flexing using the 3D EPI 1mm protocol. E, zoomed fMRI response in posterior lobe. F, fMRI response (z-stats) for hand flexing using the 3D EPI 0.8mm protocol. G, zoomed fMRI response in posterior lobe.

**
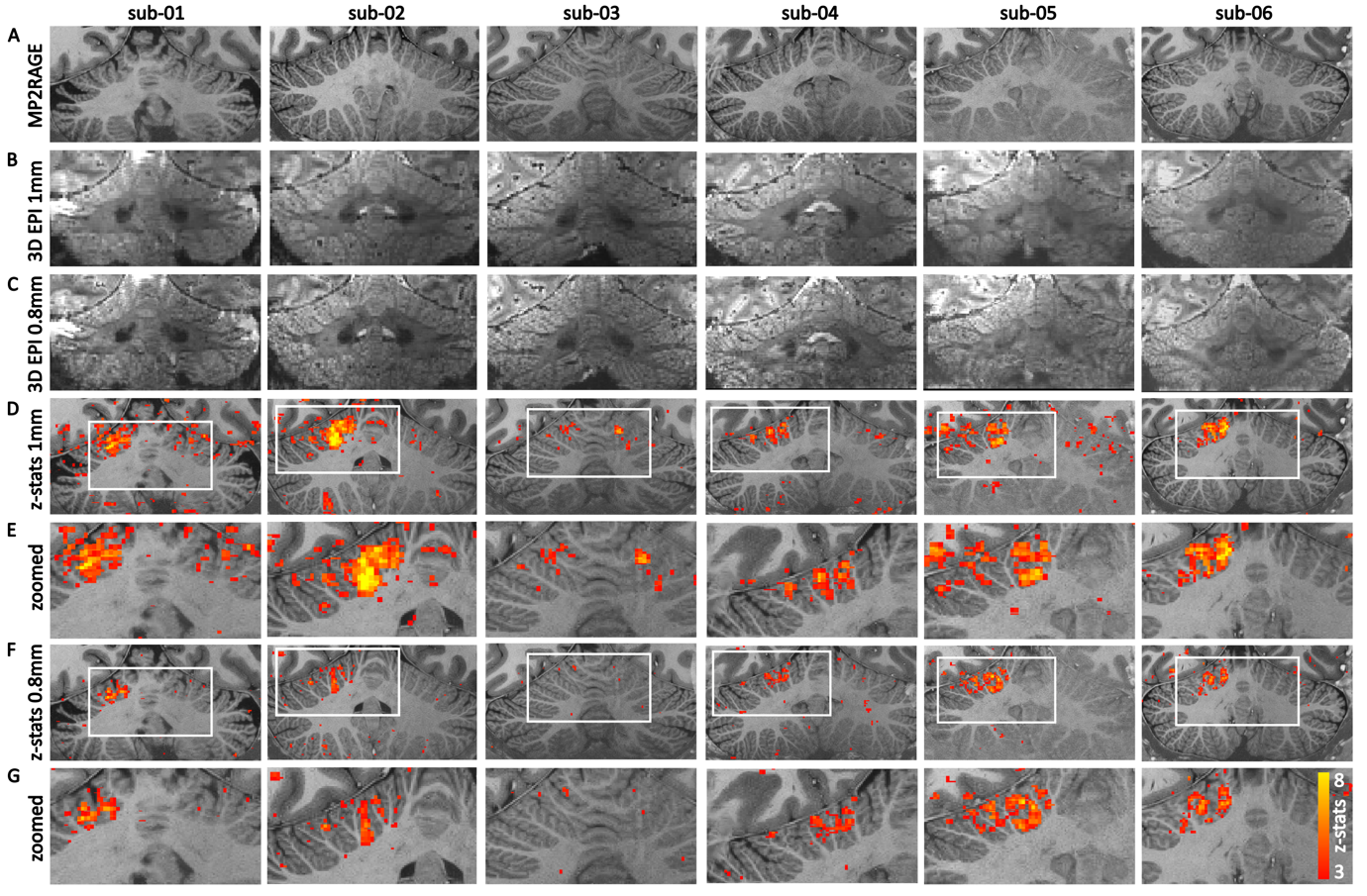
**

Table S1: T_1_ values, signal-to-noise ratio and contrast-to-noise ratio

between white (WM) and grey matter (GM) cerebellar ROIs

| Field  strength (T) | T1 (ms) | | SNR (ratio) | | CNR  WM-GM |
| --- | --- | --- | --- | --- | --- |
|  | WM | GM | WM | GM |  |
| 9.4 | 1633 | 2560 | 7.4 | 9.0 | 2.6 |
| 7 | 1139 | 2085 | 3.9 | 6.8 | 2.3 |
